# Slow GABA_A _mediated synaptic transmission in rat visual cortex

**DOI:** 10.1186/1471-2202-9-8

**Published:** 2008-01-16

**Authors:** Michael P Sceniak, M Bruce MacIver

**Affiliations:** 1 Department of Pharmacology, Case Western Reserve University, Cleveland, OH 44106, USA; 2 Department of Anesthesia, Stanford University School of Medicine, Stanford, CA 94305, USA

## Abstract

**Background:**

Previous reports of inhibition in the neocortex suggest that inhibition is mediated predominantly through GABA_A _receptors exhibiting fast kinetics. Within the hippocampus, it has been shown that GABA_A _responses can take the form of either fast or slow response kinetics. Our findings indicate, for the first time, that the neocortex displays synaptic responses with slow GABA_A _receptor mediated inhibitory postsynaptic currents (IPSCs). These IPSCs are kinetically and pharmacologically similar to responses found in the hippocampus, although the anatomical specificity of evoked responses is unique from hippocampus. Spontaneous slow GABA_A _IPSCs were recorded from both pyramidal and inhibitory neurons in rat visual cortex.

**Results:**

GABA_A _slow IPSCs were significantly different from fast responses with respect to rise times and decay time constants, but not amplitudes. Spontaneously occurring GABA_A _slow IPSCs were nearly 100 times less frequent than fast sIPSCs and both were completely abolished by the chloride channel blocker, picrotoxin. The GABA_A _subunit-specific antagonist, furosemide, depressed spontaneous and evoked GABA_A _fast IPSCs, but not slow GABA_A_-mediated IPSCs. Anatomical specificity was evident using minimal stimulation: IPSCs with slow kinetics were evoked predominantly through stimulation of layer 1/2 apical dendritic zones of layer 4 pyramidal neurons and across their basal dendrites, while GABA_A _fast IPSCs were evoked through stimulation throughout the dendritic arborization. Many evoked IPSCs were also composed of a combination of fast and slow IPSC components.

**Conclusion:**

GABA_A _slow IPSCs displayed durations that were approximately 4 fold longer than typical GABA_A _fast IPSCs, but shorter than GABA_B_-mediated inhibition. The anatomical and pharmacological specificity of evoked slow IPSCs suggests a unique origin of synaptic input. Incorporating GABA_A _slow IPSCs into computational models of cortical function will help improve our understanding of cortical information processing.

## Background

Inhibition plays an important role in visual cortical processing for receptive field formation and stimulus specificity at the local [1-3] and global network level [4-6]. *In vivo *pharmacological manipulation of inhibitory neurons alters visual cortical receptive field properties [7-9]. Understanding the kinetics of synaptic currents that give rise to inhibitory responses will be necessary to describe cortical network function and dynamics [10-13].

Within the neocortex γ-aminobutyric acid (GABA_A_) is the primary inhibitory neurotransmitter [14-21]. GABA_A _kinetics are known to be faster than GABA_B _by roughly 10-fold [14,22]. There is variability in GABA_A _subunit composition across different interneuron subtypes but the functional consequences of this subunit variability are not well known [23,24]. Different combinations of GABA_A _receptor subunits have been shown to contribute to unique inhibitory phasic and tonic response kinetics [23,25-27].

The present study of inhibition in neocortex was motivated by reports of two forms of GABA_A_-mediated inhibition in the hippocampus [28]. GABA_A _receptor mediated IPSCs in the hippocampus have fast (3–8 ms) and slow (30–70 ms) kinetic forms [28-33]. In the hippocampus, it has been shown that slow TTX insensitive spontaneous IPSCs exist, albeit infrequently, and that they can be evoked by focal electrical stimulation in the CA1 apical and basal dendritic zones, but not in the cell body layer. In contrast, fast IPSCs occur spontaneously at a high rate, but can only be evoked by micro-stimulation in the cell body layer [28,30]. Fast IPSCs are depressed by the subtype-specific GABA_A _antagonist, furosemide [34-37], while slow IPSCs are insensitive to furosemide [28,31,38]. The anatomical and pharmacological specificity argues for functionally distinct forms of GABA_A _receptor subunit combinations mediating fast and slow IPSCs.

We demonstrate here, for the first time, that GABA_A _slow currents occur both spontaneously and as evoked responses in the neocortex. Cortical GABA_A _slow responses are both quantitatively and pharmacologically distinct from GABA_A _fast responses and are evoked from anatomically distinct regions in the cortical columns.

## Results

In order to study a homogeneous population of neurons, our study focused primarily on excitatory layer 4 pyramidal neurons of visual cortex. Slow GABA_A _spontaneous synaptic currents were observed in most pyramidal neurons (35 out of 47) as well as a subset of histologically identified inteneurons (3 out of 5). Fast IPSCs were observed in all pyramidal neurons and inhibitory interneurons.

**Figure 1:**
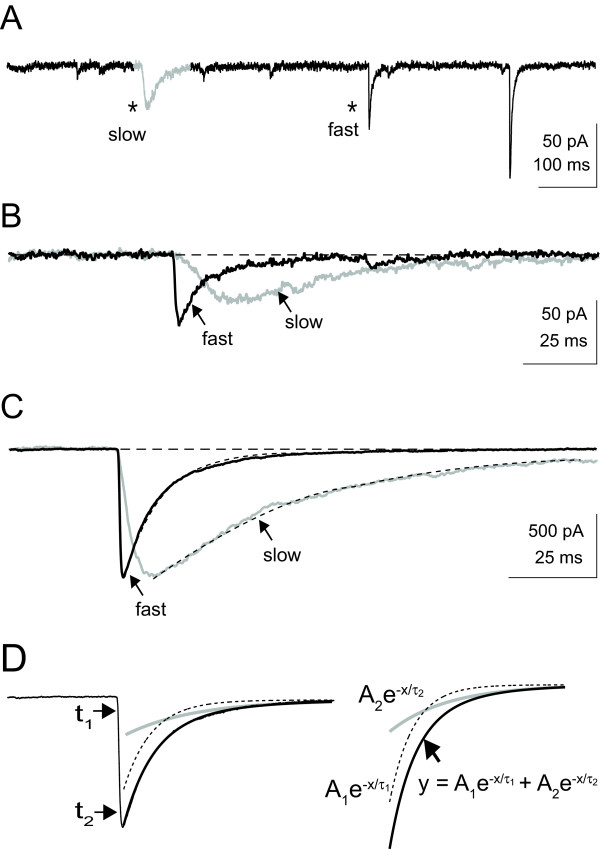
Representative whole-cell voltage-clamp recording of spontaneous activity of a neocortical pyramidal cell. A) Current recording of spontaneous IPSCs from a single cell with asterisks indicating a representative slow and fast isolated IPSC and the slow IPSC highlighted in gray. B) Overlay view of the isolated IPSCs indicated by asterisks in A. The horizontal dashed line represents the baseline current. C) Representative large amplitude spontaneous currents, showing both slow and fast events with matched amplitudes from the same cell. Dashed horizontal line represents the baseline current. The dashed line along the decaying slope represents empirical fits to the data. D) IPSC isolated events were quantified by estimating the peak amplitude from baseline, as well as the rise time and decay time constant (τ_1_). Rise times were estimated as the time between 10 and 90% of the peak amplitude (t_1 _and t_2 _respectively). Decay time constants were estimated from empirical fits with a double exponential equation. The exponential time constants (τ_1 _and τ_2_) were used as estimates of the decay time (τ_2 _> τ_1_).

Spontaneous IPSCs were recorded in rat visual cortical neurons perfused with room temperature ACSF containing 2 mM Ca^2+ ^and Mg^2+ ^(see Methods) to limit the occurrence of IPSC bursts, allowing the distinction of isolated events (Salin, 1996; Bacci, 2004). IPSCs were isolated from EPSCs through bath application of CNQX/APV (see Methods) to block glutamate-mediated events. The chloride channel blocker, picrotoxin (150 μM) completely abolished both slow and fast GABA_A _IPSCs. Across the entire population and within any single recording, sIPSCs were observed with a wide range of kinetics and amplitudes (Figure 1).

### Spontaneous IPSC parametric analysis

In order to quantify the amplitude and temporal characteristics of the spontaneously occurring IPSCs in our population of cells, single isolated IPSCs were sorted from our recordings (see Methods). All sorted IPSCs (slow and fast) were then individually analyzed to determine their amplitude from baseline and rise time (10 to 90% of peak amplitude from baseline). The decaying slope of each IPSC was fitted with a double exponential equation to determine the temporal properties of the decay to baseline (see Methods section and Figure 1D).

**Figure 2:**
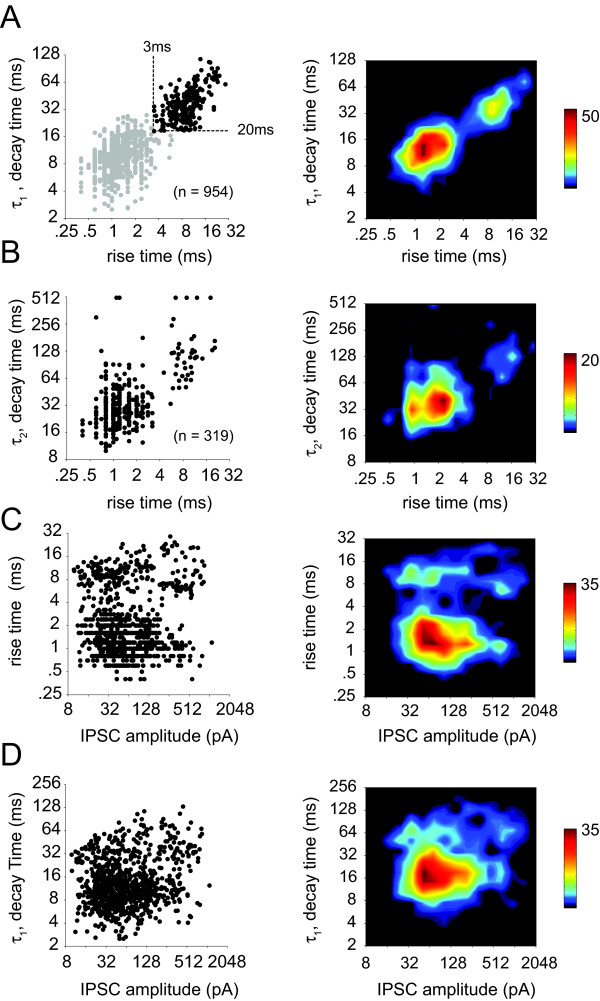
Quantitative population analysis of spontaneous isolated IPSCs. Trends in the properties of isolated IPSC amplitudes and kinetics (rise and decay time) were determined by comparing these parameters across the entire population of measured isolated IPSCs (n = 954). A) Isolated IPSC decay time constants are shown plotted vs. rise times. Two distinct populations are revealed by a correlation between rise time and decay time constants (τ_1_, first component of the double exponential). Fast IPSCs are shown in gray and slow IPSCs are shown in black. Dashed lines represent the estimated demarcation between populations, based on a slow rise time > 3 ms and decay time > 20 ms. Smoothed density plots are shown to the right. The color-coded density plots indicate that the population is bimodal with separate fast and slow sub-populations that are correlated along rise and decay (τ_1_) times. B) For those events with a significant second exponential component (|*A*_2_| > 10 pA, n = 319), τ_2 _(second decay time constant) are shown vs. rise time. Smoothed density profile to the right reveals that these parameters are correlated and cluster into two groups (fast and slow). C-D) Rise time and decay time are shown plotted against event amplitude with smoothed density profile to the right. Neither rise time nor decay time (τ_1_) was correlated with amplitude. The density profiles indicate bimodality along rise time that is uncorrelated with amplitude.

Across the population of sorted isolated IPSCs (n = 954), a parametric comparison of kinetic properties revealed two distinct populations. Rise time (> 3 ms) and decay time (τ_1 _> 20 ms) limits were used to segment the population into fast (n = 714) and slow (n = 240,) events (Figure 2A, gray and black points respectively). IPSCs with slow kinetics occurred far less frequently than fast events (0.01 Hz vs. 2.2 Hz, respectively). For events that displayed an observable second component to their decaying slope (n = 319), there was also clustering into two groups (see Methods, |*A*_2_| > 10 pA, Figure 2B). IPSC kinetics were not correlated with amplitude for either rise time or decay time (Figure 2C–D).

For a representative neuron, all IPSC events (n = 574) were sorted and analyzed to determine the quantitative kinetics and amplitudes of all events for a given cell (see Additional file 1). The mean (geometric) amplitude (53 ± 45 pA) rise time (1.1 ± 0.9 ms) and decay time constant (7.3 ± 3.1 ms) for the representative neuron was not statistically different (p > 0.05, Wilcoxon signed-rank test) from the estimates for our randomly sampled fast population (n = 714, see Table 1).

**Table 1:** Statistical summary of spontaneous IPSC kinetics and amplitudes.

	fast ‡	slow‡	p-value*
rise time	1.3 ± 0.7 ms	9.0 ± 2.5 ms	p < 0.001
decay time†			
τ_1_	10 ± 4.4 ms	36 ± 19 ms	p < 0.001
τ_2_	30 ± 42 ms	120 ± 58 ms	p < 0.001
amplitude	57 ± 100 pA	79 ± 194 pA	p > 0.05
duration	11 ± 5 ms	43 ± 22 ms	p < 0.001

†decay time constants from double exponential fit

‡standard deviation of the mean

*Student's t-test

Decay time constants τ_1 _and τ_2 _are shown for double exponential fits to the data. τ_1 _is shown for all IPSCs in the sample population (n = 954) and τ_2 _for responses with a significant second exponential (n = 319).

In order to test for possible space clamp artifacts, spontaneous IPSCs were recorded using a CsCl-based internal electrode solution (see methods), to block potassium leak currents. On average, both rise times and decay times forslow spontaneous IPSCs recorded using a CsCl-based internal solution remained within the ranges of recordings made using a KCl-based internal solution: 12.5 ± 6 ms for average slow event (n = 8) rise times and 44 ± 22 ms for slow event (n = 10) decay time constants. A random sample (n = 100) of spontaneous events recorded using CsCl-based internal solution also revealed kinetics that were similar to those recorded with KCl-based internal solution for fast spontaneous IPSCs: 1.8 ± 0.8 ms rise time and 10.4 ± 5 ms decay time constant.

**Figure 3:**
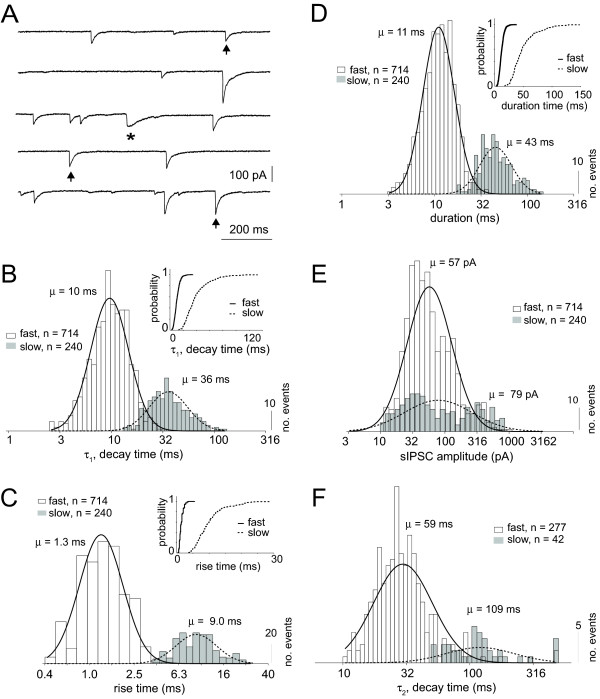
Population analysis (μ indicates geometric mean) of spontaneous isolated IPSC kinetics. The population of spontaneous isolated IPSCs was divided into two sub-populations based on the bimodal clustering in the correlation scatter plot of rise and decay time (τ_1_) (see Figure 2A). A) Representative current recording of a pyramidal neuron. Asterisk indicates the identified slow IPSC event and the vertical arrows indicate the randomly selected events. B) Population histograms and Gaussian fits of the decay time constant (τ_1_) estimates for fast (open, solid, μ = 10 ms) and slow (gray, dashed, μ = 36 ms) event sub-populations are shown plotted on the same axis. Inset plot shows cumulative distribution for the fast and slow populations (solid and dashed lines respectively). C) Population histograms of rise times for both fast (open, n = 714) and slow (gray, n = 240) events. Solid and dashed curves (fast and slow respectively) represent Gaussian fits to the distributions on a log scale (μ = 1.3 ms and 9.0 ms respectively, p < 0.01). D) Duration estimates (rise time + decay time (τ_1_) or rise time + decay time (τ_2_) for cases where |A_2_| > 10pA) are shown for fast (open, μ = 11 ms) and slow (gray, μ = 45 ms) populations. Population distributions and the cumulative distributions (inset) show minimal overlap. E) IPSC amplitudes are shown for fast (open, n = 714) and slow (gray, n = 240) events. Amplitudes for the fast and slow event groups show considerable overlap (μ = 57 pA, 79 pA respectively). F) For events with significant second decay components (|*A*_2_| > 10 pA), the second decay time distributions are shown for the fast (open, n = 277) and slow (gray, n = 42) events. Smooth curves are Gaussian fits to the fast (solid, μ = 30 ms) and slow (dashed, μ = 120 ms) population distributions.

To compare spontaneous IPSC events statistically, slow and fast events were quantitatively analyzed as separate populations (open and gray bars respectively, Figure 3). Fast and slow IPSC events (see Figure 3A) were separated based on rise time and decay time constant clustering as in Figure 2. Decay time constants (τ_1_) for both fast and slow IPSCs were skewed on a linear scale and were well fitted by a Gaussian distribution on a logarithmic scale (solid and dashed lines respectively, Figure 3B). There was significant separation of the population mean (μ indicates geometric mean, Figure 3) for the decay time constants of fast and slow events, τ_1 _(Table 1). Rise time estimates for fast and slow events also showed significant separation between the two populations (Table 1, Figure 3C). Similar separation was observed for IPSC duration (defined as rise time + decay time constant) (Figure 3D). However, amplitude estimates for fast and slow events were not significantly different across the population (Table 1, Figure 3E).

For events with an observable second component to the decaying slope of the IPSC (n = 319, |*A*_2_| > 10 pA), the second time constants (τ_2_) were log Gaussian distributed for fast (n = 277) and slow (n = 42) events (Figure 3F). There was significant separation between the mean population τ_2 _for fast and slow events (Table 1).

### Anatomical specificity of evoked GABA_A _IPSCs

**Figure 4:**
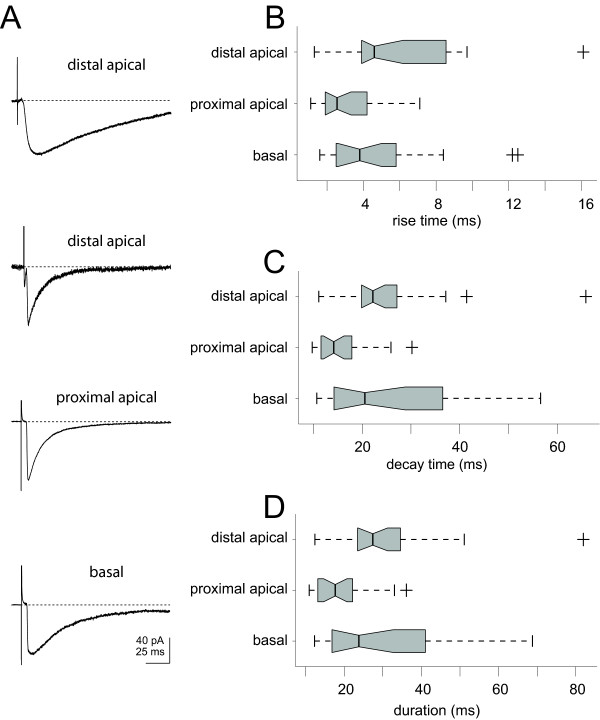
Statistical summary of anatomically classified evoked synaptic responses. A) Representative IPSC trace averages (n = 10 repeats) are shown based on the location of stimulating electrode placement: distal apical (top and second from top), proximal apical (third from top) and basal (bottom). B) Box plots show the first and third quartiles around the median with the notch signifying 95% of the median for each sample population (distal apical, n = 18; proximal apical, n = 21; basal, n = 22) and dashed lines indicate the whiskers (1.5× inter-quartile range) and crosses indicate outliers. Rise time estimates are shown summarized by the box plot for distal (median = 4.6 ms), proximal (median = 2.6 ms) and basal (median = 3.8 ms) responses. C) Decay time (τ_1_) estimates from the double-exponential fits to the IPSCs evoked from stimulation distally (median = 22 ms), proximally (median = 14 ms) or basally (median = 21 ms). D) Population summary of the total duration (rise time + decay time (τ_1_)) estimates are shown for responses evoked distally (median = 27 ms), proximally (median = 17.7 ms) and basally (median = 24 ms). Proximal stimulation produced short duration IPSCs, while distal and basal IPSCs contained a mixture of slow and fast IPSCs with greater range and variability (quartile difference (third-first) = 12 ms, 9 ms and 24 ms for distal, proximal and basal respectively).

Extracellularly evoked IPSCs were characterized based on the anatomical locus of stimulation in a sample of pyramidal cells (Figure 4A). Bipolar stimulating electrodes were positioned close to the dendrites of the recorded pyramidal neurons near (100–300 μm) the distal apical (Figure 4A, n = 18 cells), proximal apical (n = 21 cells) and basal (n = 22 cells) regions. Distal apical stimulating electrodes were placed in layer I/II directly above the dendritic axis of the cell. Basal stimulating electrodes were placed below (> 150 μm, toward the white matter) the cell body (± 100 μm from the dendritic axis). Proximal apical dendrite stimulating electrodes were placed roughly half-way between the soma and the pia off axis (100–300 μm).

Evoked IPSCs were recorded in the presence of CNQX/APV (see Methods) and the holding potential was set at rest (mean = -62 ± 4 mV). Averaged IPSCs (up to 10 repeats per cell with failures removed) were characterized for rise time, decay time and duration (Figure 4 and Table 2). Evoked responses were recorded from two locations per cell (two fixed stimulating electrodes positioned in either the proximal, distal or basal regions) in 60% of the cells and a single anatomical per cell in the remaining 40%. Slow evoked ISPCs (rise time > 3 ms and decay time, τ_1 _> 20 ms) were found predominantly in responses evoked from stimulation near the basal (9 out of 18) and distal apical dendrites (16 out of 21). Evoked IPSCs resulting from stimulation near the proximal apical dendrites (n = 22) were all fast IPSCs (rise time < 3 ms and decay time, τ_1 _< 20 ms).

**Table 2:** Statistical summary of evoked IPSC kinetics across anatomical origin of stimulation.

	rise time (ms)†		decay time, τ_1 _(ms)†		duration (ms)†	
distal apical	5.8 ± 0.6		25.2 ± 2.5		31.0 ± 3.1	
proximal apical	3.0 ± 0.6		15.5 ± 2.4		18.6 ± 3.0	
basal	5.0 ± 0.7		26.9 ± 2.7		32.0 ± 3.3	

†mean ± standard error						

ANOVA Table†	SS	df (error)	df	MS	F	Prob>F

rise time	85.5	58	2	42.7	5.12	0.0089
decay time, τ_1_	1574	58	2	787	5.95	0.0045
duration	2327	58	2	1163	5.85	0.0049

†each parameter compared across 3 groups (distal apical, proximal apical, basal)

‡ Tukey-Kramer post hoc test

Evoked IPSC were collected for stimulating electrodes placed in either the distal apical (n = 22), proximal apical (n = 18) or basal (n = 21) dendritic regions of excitatory pyramidal cells.

IPSC rise times (10 to 90% of peak amplitude) estimates were smallest for evoked responses elicited from stimulation near the proximal dendrites (median = 2.6 ms, Figure 4B). Responses evoked through stimulation in the region near the distal apical dendrites displayed rise times (median = 4.6 ms) that were significantly greater than those near the proximal dendritic region (p = 0.009, Tukey-Kramer test, ANOVA, Table 2). Evoked responses from stimulation near the basal dendrites displayed a range of rise times (median = 3.8 ms) that overlapped those from stimulation near the proximal dendritic region.

The decay time constant (τ_1_) was also characterized according to the anatomical origins of evoked stimulation (Figure 4C). Stimulation sites near the distal apical dendritic region and the basal region evoked IPSCs with decay time constants (median = 22 ms and 32 ms respectively) that were significantly slower than those evoked in the proximal apical dendritic region (median = 14 ms, p = 0.0045, Tukey-Kramer test, ANOVA, Table 2). Variability was greatest for stimulation near the distal apical and basal dendritic regions (first and third quartiles = 20 ms, 27 ms and 14 ms, 37 ms respectively) and lowest in the proximal dendritic region (first and third quartile = 12 ms and 18 ms).

Estimates of the IPSC total duration (rise time + decay time constant) were dominated by the decay time constant (τ_1_) and followed similar trends (Figure 4D). Both the distal apical and basal dendritic region stimulation estimates of duration (median = 27 ms and 24 ms respectively) were significantly longer than estimates from proximal apical dendritic region stimulation (median = 18 ms, p = 0.005, Tukey-Kramer test, ANOVA, Table 2). The distal apical and basal dendritic region stimulation estimates displayed greater range and variability (first and third quartiles = 23 ms, 35 ms and 17 ms, 41 ms respectively). Proximal apical dendritic stimulation sites produced IPSCs with the least variability (first and third quartile = 13, 22 ms).

**Figure 5:**
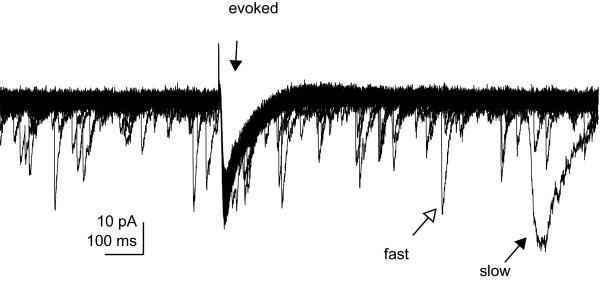
Evoked IPSCs do not inhibit the occurrence of spontaneous IPSCs Representative IPSC recording of a pyramidal cell evoked through stimulation of the input fibers within the distal apical dendrites. Slow IPSCs were evoked with 1 s separation (30 consecutive repeats). Fast spontaneous IPSCs occurred immediately before, during or following evoked ISPC stimulation. Arrows indicate specific spontaneous fast and slow events. The kinetics of the slow spontaneous events was consistent with the evoked responses. Vertical arrow marks the electrically evoked response.

Slow evoked IPSCs were observed concurrently with fast and slow sIPSCs (n = 5 cells). During periods of evoked stimulation with pulses delivered near the distal dendrites, fast and slow sIPSCs were observed (Figure 5). Evoked IPSCs and sIPSCs did not appear to interact during distal dendritic region stimulation. Fast and slow sIPSCs were observed within the period immediately preceding and following evoked stimulation, as well as overlapping evoked IPSCs (Figure 5). Neocortical evoked slow IPSCs do not appear to suppress sIPSCs with either slow or fast kinetics.

### Furosemide action on GABA_A_-mediated IPSCs

**Figure 6:**
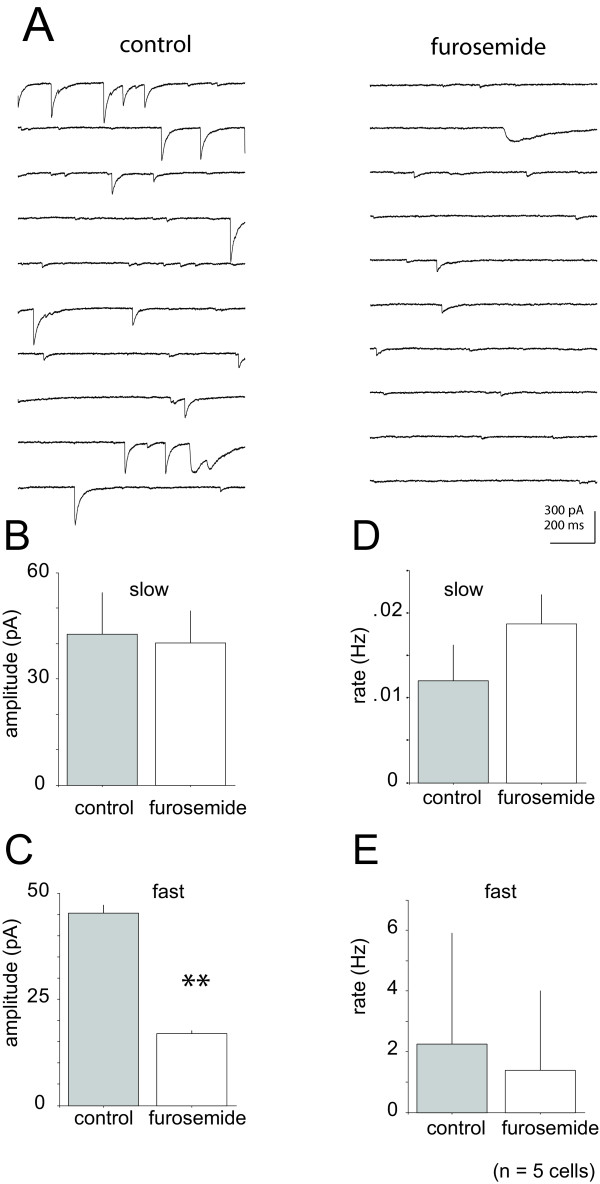
Furosemide selectively depressed GABA_A _fast spontaneous events. Population (n = 5 cells) summary for spontaneous IPSCs in control and furosemide treated conditions. A) Representative recording or spontaneous events in control and furosemide treated conditions. B) The average amplitude of slow sIPSCs was not significantly different between control and furosemide treated conditions (mean = 43 ± 12 pA and mean = 40 ± 9 pA respectively). C) The amplitude of sIPSCs was significantly reduced (mean control = 45 pA, mean treated = 16 pA) with treatment of furosemide (** indicates p < 0.001, Wilcoxon signed-rank test). D-E) The frequency of spontaneous IPSCs in the presence of furosemide was not significantly reduced for fast (mean = 2.2 ± 3.7 Hz, mean = 1.4 ± 2.6 Hz, control and treated respectively) or slow events (mean = 0.012 ± 0.004 Hz, mean = 0.019 ± 0.0034 Hz, control and treated respectively).

Furosemide, the subunit selective GABA_A _receptor blocker, selectively depresses GABA_A _fast IPSCs in the hippocampus, while leaving GABA_A _slow responses unaffected [28,31,32,38]. The effects of furosemide were tested on spontaneous currents from layer 4 cortical pyramidal neurons (Figure 6A). Furosemide (1 mM) had no significant effect on the amplitude of slow IPSCs compared to control (mean = 42 ± 12 pA, mean = 40 ± 9 pA, control and treated conditions respectively) observed in our sample population (p > 0.05, Wilcoxon test, n = 5, Figure 6B). In contrast, the amplitude of fast IPSCs was significantly reduced (65%) in the presence of furosemide (mean = 45 ± 1.9 pA, mean = 16 ± 0.7 pA, control and treated respectively) compared to control (p < 0.01, Wilcoxon signed-rank test, Figure 6B). IPSC frequency was not significantly reduced in the presence of furosemide for either fast (mean = 2.2 ± 3.7 Hz, mean = 1.4 ± 2.6 Hz control and treated respectively) or slow events (mean = 0.012 ± 0.004 Hz, mean = 0.019 ± 0.0034 Hz control and treated respectively, see Figure 6D&E).

**Figure 7:**
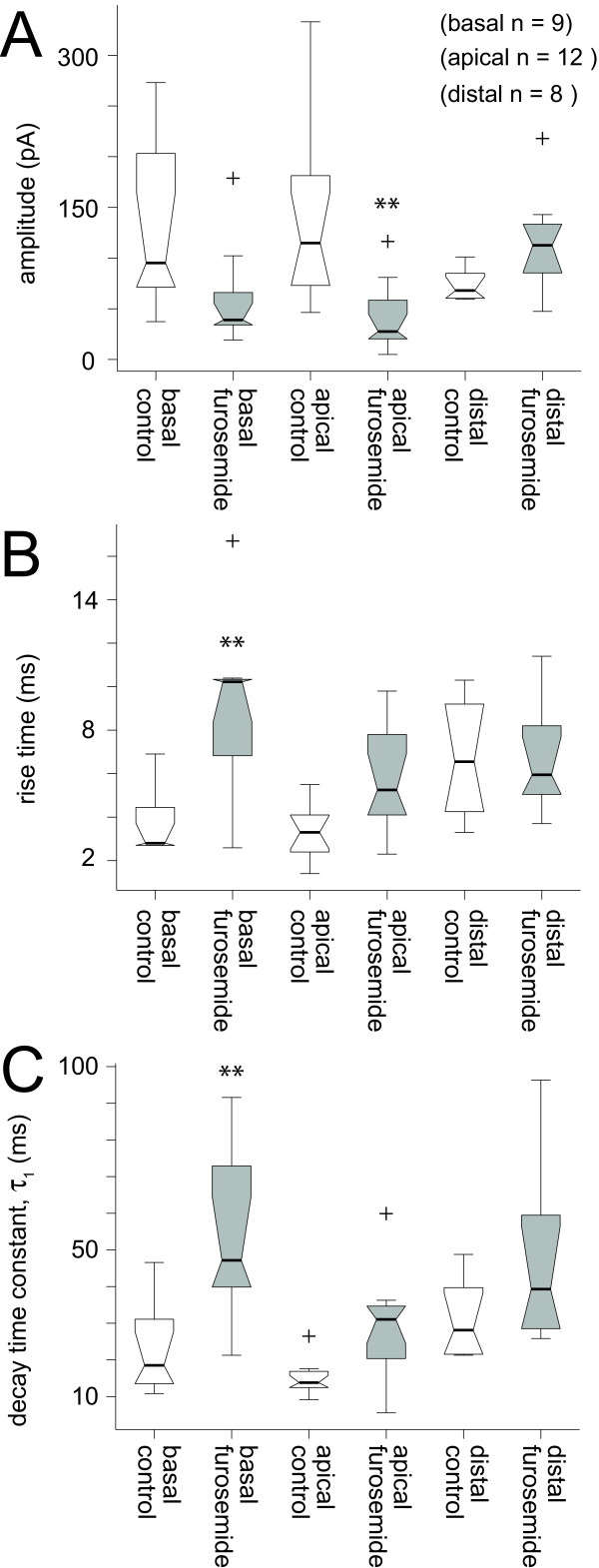
Effects of furosemide on evoked IPSCs based on anatomical origins of stimulation. Controls vs. furosemide treated (1 mM) conditions are shown in box plot form. Furosemide responses are shown in gray and control groups as open boxes. Evoked IPSC response amplitude, rise time and decay time constants are shown for stimulation of the basal (basal, n = 9), proximal apical (apical, n = 12) and distal apical (distal, n = 8) dendritic regions. Significance (p < 0.01) is indicated by ** (MANOVA and ANOVA factorial, see Table 4). Boxes span the first and third quartiles with medians indicated by thick center line and notch. A) Evoked IPSC amplitudes were reduced in the presence of furosemide compared to control for the stimulation sites in the region of the basal and proximal apical dendrites, but not the distal apical dendrites (see Table 3 for quantification). B) Rise time estimates were greater in the presence of furosemide for stimulation sites near the basal and proximal apical dendrites but not the distal apical dendrites. C) On average, the decay time constants (τ_1_) for evoked IPSCs were significantly greater with furosemide treatment than control conditions for stimulation sites near the basal dendrites. Decay time constants were not significantly different for responses evoked through stimulation of the distal apical dendrites in the presence of furosemide compared to control.

Evoked responses were recorded from extracellular stimulation of the distal and proximal apical as well as the basal dendritic region of pyramidal cells in control and furosemide (1 mM) treated conditions (n = 29, Table 3, Figure 7). Furosemide significantly depressed (p < 0.01, MANOVA, Table 4) evoked fast IPSC responses from stimulation sites originating in the proximal apical dendritic region (median = 114 pA and 28 pA for control and furosemide condition respectively; Table 3, Figure 7A). Responses evoked from stimulation near the basal dendritic region showed a trend toward depression from application of furosemide (median = 95 pA and 39 pA, control and furosemide respectively). Furosemide did not significantly alter evoked response amplitudes (median = 68 pA and 113 pA, control and furosemide respectively) originating in the distal apical dendrites (p = 0.05, Table 4, Figure 7A).

**Table 3:** Summary of furosemide effects on synaptic responses vs. anatomical location of stimulation site.

	control	furosemide	n
amplitude			
basal	95 (71, 203) pA	39 (34, 66) pA	9
apical	114 (73, 181) pA	28 (20, 58) pA	12
distal	68 (60, 85) pA	113 (85, 133) pA	8
rise time			
basal	2.8 (2.7, 4.5) ms	10.2 (6.9, 10.4) ms	9
apical	3.3 (2.4, 4.1) ms	5.3 (4.1, 7.8) ms	12
distal	6.6 (4.3, 9.2) ms	6.0 (5.1, 8.2) ms	8
decay time constant, τ_1_			
basal	18.5 (13.5, 31.0) ms	47.2 (40.0, 73.0) ms	9
apical	13.8 (12.5, 17.0) ms	31.1 (20.4, 34.7) ms	12
distal	28.1 (21.6, 39.7) ms	39.3 (28.5, 59.5) ms	8

Median values for control and furosemide groups are shown with the first and third quartiles indicated in parentheses. Stimulation sites were grouped according to their proximity to the basal (basal), proximal apical (apical), or the distal apical (distal) dendritic region of each pyramidal neuron. The decay time constants were estimated from the double exponential fits to the IPSC decaying response phase.

Treatment with furosemide resulted in prolonged rise times for evoked IPSCs for responses evoked from stimulation of the basal (median = 2.8 ms and 10.2 ms, control and furosemide respectively, Table 3) and proximal apical (median = 3.3 ms and 5.3 ms, control and furosemide respectively) dendritic regions (Table 3, Figure 7B). The rise times were not significantly (p > 0.05, ANOVA factorial) altered for responses evoked through stimulation of the distal apical dendritic regions (median = 6.6 ms and 6.0 ms, control and furosemide respectively, Table 3).

Evoked IPSCs displayed increased decay time constants in the presence of furosemide for stimulation sites originating near the basal and proximal apical dendritic regions (Figure 7C, Table 3). Decay time constants were significantly longer (p < 0.01, ANOVA factorial) in the presence furosemide (median = 47.2 ms) versus control (median = 18.5 ms) for stimulation sites located near the basal dendrites (Figure 7C, Table 3). Evoked IPSC responses showed a trend toward increased decay time constant duration in the presence of furosemide (median = 31.1 ms) compared to control (median = 13.8 ms) for stimulation sites near the proximal apical dendrites (Figure 7C, Table 3). Stimulation sites located near the distal apical dendrites produced IPSCs with no significant difference (p > 0.05, ANOVA factorial, MANOVA, Table 4) in the median decay time constants in control or furosemide treated condtions (median = 28.1 ms and 39.3 ms, control and furosemide respectively).

**Table 4:** MANOVA: Furosemide effects vs. stimulation site.

	dfW	dfB	dfT	Λ	p	
basal	16	1	17	0.46	0.009**	
proximal apical	22	1	23	0.45	0.001**	
distal	14	1	15	0.52	0.05	

One-way multivariate analysis of variance (MANOVA) table for the effects of furosemide on evoked synaptic responses resulting from stimulation at sites near the basal, proximal apical, and distal apical dendritic regions. Degrees of freedom are shown for each stimulation category for within-group sum of squares and cross-products (dfW), between-group sum of squares and cross-products (dfB) and the total sum of squares and cross-products (dfT). Wilk's Λ test statistics are shown for each stimulation category along with the resulting p-value (** indicates significance < 0.01).

Furosemide only partially depressed many of the evoked responses and the residual components often displayed slower kinetics than the control condition. In other cases furosemide completely suppressed the evoked response. Increasing the stimulus intensity in the presence of furosemide revealed an evoked IPSC with slow kinetics even in cases (n = 8) where the responses were completely suppressed (prior to stimulus intensity increase). Picrotoxin (150 μM) completely suppressed all evoked responses. Furosemide appeared to selectively depress the fast component of responses, revealing a slow IPSC that contributed to most evoked responses. This occurred for responses evoked through distal and proximal apical dendritic and basal dendritic region stimulation.

## Discussion

Our study shows that both evoked and spontaneous GABA_A_-mediated slow IPSCs occur in neocortex. Slow GABA_A_-mediated IPSCs display quantitatively similar kinetics properties for rise time and decay time (τ_1_) as those observed in the hippocampus [28,29,31,32,38-40]. Spontaneous GABA_A _slow IPSCs in the neocortex occurred infrequently compared to GABA_A _fast events. The low frequency of occurrence of spontaneous slow GABA_A_-mediated IPSCs could explain the lack of previous reports of this slow synaptic inhibition in neocortex.

Inhibitory interneurons within the neocortex form a highly interconnected and heterogeneous population of cells that appear to segregate into unique groups both morphologically and functionally [1,20,23,24,41-43]. Two well described neocortical interneuron subtypes, fast-spiking (FS) and low-threshold-spiking (LTS) cells, can be distinguished by their unique synaptic kinetics [25,27,41,42,44,45]. The IPSCs from FS and LTS cells have fast synaptic current rise times, but LTS cells have slower decay time constants. The slow GABA_A_-mediated currents presented here display even slower rise times and decay time constants than the IPSCs reported for FS and LTS cells. Perhaps GABA_A _slow IPSCs also arise from of a unique subtype of interneuron.

It is unlikely that slow GABA_A _IPSCs are merely a result of filtering from the cable properties of dendrites. We have shown here that pure fast as well as slow GABA_A_-mediated IPSCs were preferentially evoked through electrical stimulation of the distal apical dendritic zones (superficial layer 1/2) of layer 4 pyramidal neurons. Previous reports have shown that, unlike EPSCs, IPSCs evoked in distal apical dendrites of pyramidal neurons show little effects of kinetic slowing from dendritic cable properties [28,40,46-50]. Also we have observed similar time constants of IPSCs recorded with either CsCl or KCl internal solutions. CsCl is known to block K+ channels and increase the signal to noise ratio for distal IPSCs. In addition, furosemide failed to block distally evoked evens, suggesting a distinct pharmacological action based on the anatomical origin of stimulation.

Slow GABA_A_-mediated IPSCs observed in the neocortex are pharmacologically distinct from fast GABA_A _IPSCs. A GABA_A _subtype-specific antagonist, furosemide [34-37], did not alter GABA_A _slow IPSCs, while it markedly attenuated spontaneous GABA_A _fast responses amplitudes (~90%). GABA_A_-mediated IPSCs observed within the hippocampus show similar furosemide selectivity [28]. The differential sensitivity of fast and slow components to furosemide suggests that slow and fast responses in neocortex have unique GABA_A _receptor subunit compositions. GABA receptors containing either α1 or α4 subunits display the fastest decay time constants [51,52]. GABA receptors containing either α2 [51], α3 [53], or α5 [52] have slower decay kinetics than either α1 or α4. Furthermore, furosemide has a 50-fold greater selectivity at α4βγ2 GABA_A _receptors, which have fast kinetics, compared to other isoforms [54]. Blocking these fast-decaying receptors would yield a pool of slower (α2, α3, α5) or similarly slow decaying (α1) receptors.

It has been shown previously that furosemide nonspecifically blocks the K^+^/Cl^- ^cotransporters NKCC1 and KCC2 [55-57]. Blocking Cl^- ^cotransporters would increase internal Cl^- ^concentrations and likely decrease the time constants of IPSCs by increasing membrane resistance. However, we observed a time constant increase in the presence of furosemide. Nonspecific effects on Cl^- ^cotransporters are also unlikely to affect synaptic responses in our preparation, because the chloride gradient was already reversed with a KCl-based internal solution (see Methods).

Some evoked and spontaneous responses were composed of a combination of slow and fast components. Similar combined fast and slow components of GABA_A_-mediated IPSCs have also been observed in piriform cortex [58]. The slower second components of evoked IPSCs were proposed to result from activation of GABAa slow receptors. However, spontaneously occurring slow IPSCs have not been shown in piriform cortex.

At least one-third of spontaneous events in our study contained a significant slow second component decay time constant as revealed with double-exponential fits. Application of furosemide revealed a residual response component with slow kinetics in evoked IPSCs for many cells. The decay time constants (τ_1_) of the residual currents were consistent with GABA_A _slow currents.

Anatomical comparison revealed that IPSC responses with purely slow components were evoked in layer 4 pyramidal neurons through microstimulation of either the distal apical (layers 1/2) or basal (layers 5 or 6) dendrites but not the proximal apical dendrites. In contrast, fast IPSC responses were evoked with near-threshold stimulation throughout the dendritic arbor. In the presence of furosemide, slow IPSCs were revealed in otherwise fast IPSC evoked responses at all locations of dendritic stimulation (distal, proximal and basal). IPSCs evoked through stimulation of distal apical dendrites were also not depressed by furosemide. The presence of slow IPSC components in the evoked responses at proximal apical dendrites with application of furosemide suggests that GABA_A _slow responses occur throughout the dendritic length but are masked by stronger fast responses. This might result from a higher density of GABA_A _receptor subtypes responsible for fast responses at these locations. The exact location of synapses evoked through our stimulation might not correspond to our stimulation sites. Because the synapses of any particular stimulation site might terminate some distance from the origin of stimulation, our anatomical classification cannot distinguish between the location of cell bodies versus synaptic inputs.

Slow spontaneous IPSCs might result from a unique population of inhibitory synapses containing GABA_A _receptors composed of subunits with only slow kinetics [26,28,39]. Another possibility is that perisynaptic tonic receptors are activated by spillover of GABA from nearby fast phasic synapses without activating GABA_A _receptors that contribute to fast responses [29,33,39,59]. The low frequency of occurrence of these slow spontaneous events might result from the unique anatomical arrangement required for such an event to occur. Both mechanisms have been suggested previously to explain GABA_A _slow responses observed in the hippocampus [28,31,32,59]. At this time, it is not possible to distinguish between activation of synapses with purely slow GABA_A _receptors versus spillover. Selective agonists and antagonists for both types of receptors or specific knockout/knockin genetic models will be needed for future studies [26].

The results presented here focused primarily on inhibitory synaptic inputs to excitatory pyramidal neurons. However, we observed spontaneous slow GABA_A _IPSCs in neocortical interneurons as well. Because of the diversity of subtypes of inhibitory neurons, a systematic analysis of each subtype will be required to make meaningful statistical arguments for each subtype and is beyond the scope of this study [23,24]. GABA_A _slow IPSC responses evoked in inhibitory neurons may provide a fruitful direction for future investigations.

## Conclusion

Inhibitory synaptic inputs of neocortical neurons are an important component of feedforward and feedback processing [1-3,9,60]. It has been shown that visual evoked field potentials display differential sensitivity to GABA_A _and GABA_B _blockade for afferent feedforward components versus long-range feedback interactions [6]. It will be important to determine what role slow GABA responses have in specific aspects of visual feedforward or feedback processing. New genetic and molecular tools will be necessary to study these mechanisms *in vivo*.

Intrinsic network oscillations also appear to be controlled by inhibitory inputs [38,61]. GABA_A _slow IPSCs displayed durations ranging from 30 to 125 ms; therefore, slow GABA_A_-mediated IPSCs might play a role in controlling alpha or beta rhythm (8 to 30 Hz) activity [25,62-65]. Incorporating GABA_A _slow IPSCs into computational models of cortical function will help improve our understanding of cortical information processing.

## Methods

### Electrophysiology

All procedures and protocols were approved by the Institutional Animal Care Committee at Stanford University and adhered to guidelines published by the National Institutes of Health. All experiments were performed on rat brain slices dissected from the visual cortex of young (P16-P28) male Long-Evans rats (Charles River Laboratories, Wilmington, MA). The preparation of rat neocortical brain slices was identical to that described in Sceniak and MacIver [66].

Briefly, parasagittal brain slices were cut in cold (4°C) oxygenated (95% O_2_, 5% CO_2_) artificial cerebral spinal fluid (ACSF) into 350 μm thick sections. The brain slices were then placed in room temperature ACSF containing the following: NaCl, KCl, MgSO_4_, NaH_2_PO_4 _NaCHO_3_, dextrose and CaCl_2 _in the following mM concentrations: 124, 3.5, 2, 1.25, 26, 10 and 2 [25,66,67]. All recordings were conducted at room temperature with the same ACSF. Slices were visualized with an upright microscope (Zeiss Axioskop, Germany), using a water immersion objective (40×, Zeiss) with near infrared illumination and a CCD camera (COHU, San Diego, CA).

Whole-cell patch clamp recordings were amplified with a Multiclamp 700A patch clamp amplifier (Axon Instruments, Foster City, CA). Voltage and current traces were sampled at 10 kHz. Data acquisition was controlled using the commercially available software package, pCLAMP 9.0 (Axon Instruments, Foster City, CA). Recording electrodes were filled with a KCl-based internal solution containing, in mM 100 KCl, 10 EGTA, 40 HEPES, 5 MgCl_2_, 2 Na_2_ATP and 1.5 Na_2_GTP (pH 7.3 and osmolarity 290–295 mOsm).

In a subset of cells (n = 4), artifacts from space clamp were tested by recording spontaneous IPSCs with a CsCl-based internal electrode solution. Internal patch pipette solution contained, in mM, 140 CsCl, 2 MgCl_2_, 40 HEPES, 10 EGTA, 2 Na_2_ATP, 1.5 Na_2_GTP (pH 7.3 with CsOH and 290–295 mOsM).

Synaptically evoked responses were elicited using bipolar stimulating electrodes fabricated with either theta-glass pipettes or pairs of epoxy-coated tungsten microelectrodes encased in a single barrel glass pipette (Harvard Apparatus, Holliston, MA). Stimulating electrodes of either theta-glass or tungsten (10–30 μm or 5 MΩ tips respectively) were positioned near the dendritic field of targeted pyramidal cells (100–300 μm from the dendritic axis). Stimulus intensity was optimized for each cell to produce monosynaptic near-threshold amplitude IPSCs (0.005 mA to 1.0 mA, 0.1 ms pulse). Minimal responses were determined to be near-threshold level when decreasing the stimulus intensity resulted in failures < 50% of the time and the response no longer decreased in amplitude. Minimally evoked responses also displayed amplitudes that were similar to spontaneously occurring events. Synaptically evoked IPSC responses were recorded with the cell clamped at its resting membrane potential (-65 ± 5 mV) to avoid imposing additional leak currents within the cell.

All EPSCs were blocked by bath application of (±)-2-amino-5-phosphonopentanoic acid (APV) (100 μM) and 6-cyano-7-nitro-quinoxaline-2, 3-dione (CNQX) (17.2 μM) to block NMDA and AMPA receptor-mediated synaptic currents respectively. Spontaneous IPSCs were recorded with each cell voltage-clamped at its resting membrane potential using KCl-based internal solution that reversed the driving force of the chloride currents and increased the amplitude of small IPSCs.

Drug treatment with furosemide (1 mM) and picrotoxin (150 μM) were always preceded by data collection of at least five runs with 10 repeats over a 20 m period to determine baseline response. Drugs were allowed to perfuse into the tissue for at least 15 m with responses monitored during this period, in order to determine response stability. Next, drug responses were collected with a minimum of 5 runs with 10 repeats over 15 m for each condition. Responses were analyzed for the last 3 runs (10 repeats each) of each condition during the most stable periods of drug perfusion and control conditions.

### Data collection and analysis

Average electrode series resistance ranged from 10–20 MΩ after break in. Whole-cell recording seal impedance ranged from 1–3 GΩ. Average resting membrane potentials were -65 ± 5 mV. Recordings with seals less than 1 GΩ or resting membrane potentials greater than -55 mV were not included in the analysis. In voltage-clamp experiments, access resistance was monitored using a 20 ms voltage step deviation from the holding potential and repeated throughout the duration of the recording.

Spontaneous current recordings were collected and digitally stored (30 s long continuous current recording, 10 repeats or more, 10 kHz sampling). IPSC events were automatically detected through a computer algorithm (custom routines written in MATLAB) that identified peaks that were negatively shifted at least 2 standard deviations from the baseline. Mean baseline noise floor was 12 ± 5 pA. Slow events were selected initially based on visual inspection. A control group of IPSCs was randomly selected from all recordings to compare to the sampled slow IPSC population. The control IPSC events were randomly selected from the recordings that contained the visually identified slow IPSCs as well as from randomly selected recordings that lacked slow IPSCs.

For a representative neuron all events (greater than 2 SD above baseline) were analyzed and the kinetics compared with our random sample of events from all cells. The kinetic and amplitude properties were quantitatively similar for the single cell estimates with all events included and the random sample population across all cells (see Results).

Isolated spontaneous and evoked IPSCs were fitted with a double exponential equation to determine the decay time constant after the IPSC peak. The double exponential empirical function was of the following form:

$$y=A_{1}e^{-x/\tau_{1}}+A_{2}e^{-x/\tau_{2}}.$$

The parameters *A*_1 _and *A*_2 _represent the amplitude of the two components. The equation was fitted to the data using a constrained nonlinear least squares optimization routine (fmincon, Matlab). Nonlinear constraints were used such that τ_2 _was greater than τ_1_. Rise times were estimated as the time from 10 to 90% of the peak amplitude (t_1 _and t_2 _respectively) from baseline (see Figure 1). For fast events the rise times show binning at the sampling rate (see Figure 2A), because the estimates were not fitted or interpolated.

IPSC events were classified as slow or fast based on the rise time and decay time kinetics [47]. Correlations of rise time and decay time revealed a clustering into two unique populations. The demarcation between the fast and slow population was determined from the local minimum in the intensity plot of the correlation between rise time and decay time. Slow events were defined as IPSCs with rise times greater than 3 ms and decay time constants greater than 20 ms. Events that were beyond the constraints of the slow classification were considered fast events (see Figure 2A).

All data analysis and statistical tests were performed using MATLAB Release 12 (Mathworks, Natick, MA). All statistics are expressed as the mean ± standard deviation unless otherwise stated. Statistical significance of data from control and drug treated groups was determined using the Student's *t *test or Wilcoxon signed-rank test, for cases where the data was not normal on a linear scale. One-way multivariate analysis of variance (MANOVA) was used to compare differences across groups of multivariate data. Multi-way factorial ANOVA was used to compare differences from three or more independent groups. Time locked current and voltage traces were averaged in Matlab to produce mean evoked IPSCs.

### Histology

Excitatory pyramidal neurons were targeted for all recordings. However, histological reconstructions revealed that a subset of the cells (5 out of 52) were inhibitory aspiny interneurons. All electrically evoked synaptic responses were recorded from excitatory layer 4 pyramidal neurons. In some cases the histological analysis was not successful and therefore, it is possible that layer 4 and 5 neurons have been combined. Cell body morphology and spiking responses were consistent across all pyramidal cells.

In order to perform histological reconstructions the internal solution contained 0.5% to 1% neurobiotin (Vector Laboratories, Burlingame, CA). Slices were processed with the Elite VectaStain ABC kit (Vector Laboratories, Burlingame, CA) according to the protocol described by Hamam and Kennedy [68]. Stained slices were mounted wet with Vectashield mounting medium (Vector Laboratories, Burlingame, CA). Digital images were reconstructed using Adobe Photoshop (Adobe Systems Incorporated, San Jose, CA) to determine cell morphology and the presence of dendritic spines.

### Animals/Chemicals

All rats were obtained from Charles Rivers Laboratories (Wilmington, MA). Chemicals for the ACSF were reagent grade or better and obtained from J. T. Baker (Philadelphia, PA) or Sigma-Aldrich (St. Louis, MO).

## Supplementary Material

Additional file 1 IPSC analysis of all events for a representative cell. For a representative neuron, all isolated IPSC events (n = 461) were sampled and fitted to determine the amplitude, rise time and decay time. The vertical arrows indicate the population geometric mean for the amplitude (52.6 ± 44.6 pA) rise time (1.1 ± 0.9 ms) and the decay time constant (7.3 ± 3.1 ms). The average rate was 1.9 Hz across all sampled events (30 s long data records, 8 repeats, 240 s total). These measures are quantitatively similar to our random sample of fast IPSC events. Click here for file
